# Joint variation and ZhuYin dataset for Traditional Chinese document enhancement

**DOI:** 10.1038/s41597-024-04146-7

**Published:** 2024-11-27

**Authors:** Shi-Wei Lo, Hsiu-Mei Chou, Jyh-Horng Wu

**Affiliations:** National Center for High-Performance Computing, Hsinchu, Taiwan

**Keywords:** Information technology, Scientific data

## Abstract

Digital documents play a crucial role in contemporary information management. However, their quality can be significantly impacted by various factors such as hand-drawn annotations, image distortion, watermarks, stains, and degradation. Deep learning-based methods have emerged as powerful tools for document enhancement. However, their effectiveness relies heavily on the availability of high-quality training and evaluation datasets. Unfortunately, such benchmark datasets are relatively scarce, particularly in the domain of Traditional Chinese documents. We introduce a novel dataset termed “Joint Variation and ZhuYin dataset (JVZY)” to address this gap. This dataset comprises 20,000 images and 1.92 million words, encompassing various document degradation characteristics. It also includes unique phonetic symbols in Traditional Chinese, catering to the specific localization requirements. By releasing this dataset, we aim to construct a continuously evolving resource explicitly tailored to the diverse needs of Traditional Chinese document enhancement. This resource aims to facilitate the development of applications that can effectively address the challenges posed by unique phonetic symbols and varied file degradation characteristics encountered in Traditional Chinese documents.

## Background & Summary

In recent years, with the advancement and widespread application of science and technology, the digitization of documents has become an inevitable trend. One of the primary objectives of this digitization process is to recover a clean version of degraded documents. However, numerous challenges often arise during the digitization of paper documents, including dirt, discoloration, blurring, distortion, watermarks, and other degraded factors, all of which can adversely affect the quality of the digitized output. Therefore, enhancing the digital quality of paper documents has emerged as a critical issue^1,2^. Various techniques and methods exist for noise removal while digitizing paper documents. Traditional methods include threshold processing, masking, and morphological processing, among others. While conventional image processing can effectively remove some noise, it may not always be effective for more complex and diverse degraded documents, such as those with handwriting and stains^3,4^.

Today, many researchers have turned to artificial intelligence (AI) methods to tackle the challenges associated with document enhancement. Convolutional Neural Networks (CNNs) have emerged as one of the most popular and practical AI technologies, widely utilized in fields such as image recognition, image processing, and natural language processing. Within the context of image-to-image frameworks, deep convolutional neural networks have demonstrated significant success in document enhancement tasks. Image-to-image frameworks leverage paired degraded images and ground truth images for end-to-end training, allowing for the direct transformation of input images into clean images. For instance, autoencoders and variational autoencoders (VAEs)^5–7^, as well as approaches based on Generative Adversarial Networks (GANs)^8–10^, Transformer-based methods^11^, and diffusion-based techniques^12^, have all demonstrated promising results in this domain.

The performance of these deep learning methods, as reported across various datasets, consistently demonstrates improvement, showcasing their capability to restore degraded documents to their ideal states. However, the effectiveness of neural network models relies heavily on training with large quantities of high-quality datasets. The successful advancements in research are predominantly based on datasets primarily composed of English documents^13–18^.

While studies are focusing on various aspects of Chinese document processing, such as the restoration and cleaning of ancient Chinese texts^19,20^, historical Chinese character recognition^21^, adaptive recognition based on simplified Chinese characters^22^, generation of individual Chinese characters^23^, and the creation of traditional Chinese datasets for scene text recognition^24^, there remains a notable gap in the availability of datasets specifically tailored for document enhancement tasks in Traditional Chinese.

Furthermore, in real-world scenarios, documents often undergo varying degrees of degradation, such as hand-drawn annotations, color highlights, or scribbles, which can impact the digitization of paper documents. Moreover, digitizing Chinese documents presents unique challenges. Chinese characters typically consist of numerous strokes and complex shapes, and accompanying phonetic symbols are often offered alongside the characters for pronunciation and reading purposes. Chinese characters are ideographic rather than phonetic, and complex strokes characterize their structures. Therefore, native Chinese speakers and beginners learning Chinese typically begin by mastering a phonetic system for Chinese characters, such as Zhuyin symbols or Hanyu Pinyin, to learn the character. While radicals in Chinese characters can provide clues to pronunciation, they are not always systematic. Phonetic annotations are essential for accurate pronunciation and facilitate independent reading of unfamiliar characters. Additionally, phonetic symbols can be substituted when encountering characters one cannot write. Thus, for native Chinese-speaking children and beginners learning Chinese, a phonetic system for learning Chinese character pronunciation is necessary.

In the Optical Character Recognition (OCR) process, these Zhuyin phonetic symbols are often erroneously classified as noise, making it challenging to distinguish them from other characters^25^. Moreover, the lack of datasets designed explicitly for enhancing Traditional Chinese documents has been a bottleneck, prompting the initiation of this study to propose the first dedicated training set for Traditional Chinese document enhancement.

The success of computer vision models largely hinges on the support of large-scale annotated real-world images. However, the manual processing required to pre-clean degraded documents into clean ground truth samples and the difficulty in collecting a diverse range of document degradation patterns poses significant challenges. Additionally, using actual documents as sources presents concerns regarding privacy protection and the burden of pre-digitization, cropping, and other processing tasks. Such annotated datasets are challenging to manage in document enhancement applications.

To aid document enhancement, synthetic data generation, management, and annotation offer an economically efficient means to swiftly achieve the data diversity necessary for improving model performance at different stages. This study proposes a large-scale synthetic dataset of degraded Traditional Chinese documents, paired with clean ground truth textual content, termed the “Joint Variation and ZhuYin Dataset (JVZY).” This dataset comprises paired images totaling 20,000 and 1.92 million words, featuring various document degradation characteristics and annotating Zhuyin phonetic symbols unique to Traditional Chinese, further catering to its localization-specific requirements.

## Methods

In this section, we provide a detailed overview of all the steps involved in creating JVZY to ensure the quality, diversity, and efficiency of the synthetic dataset. The generation of synthetic documents is automated and image-based, simulating printing text on paper by drawing foreground text pixels on a canvas and adding various artificial degradation effects.

**Fig. 1:**
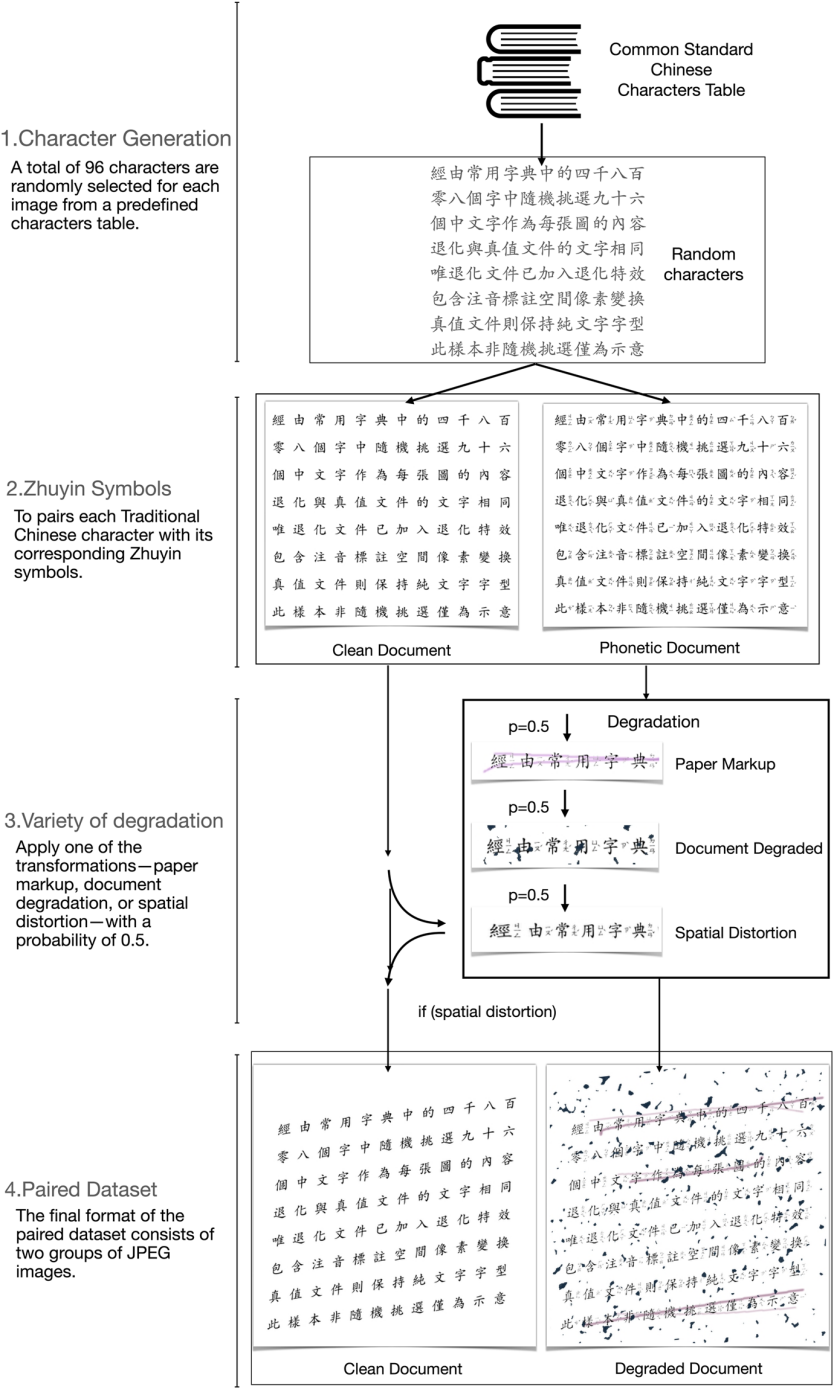
The workflow for synthesizing the JVZY dataset consists of four stages: 1. Chinese character generation, 2. Zhuyin symbol annotations, 3. composed variety of degradation, and 4. resulted in paired dataset.

Figure 1 illustrates the complete implementation process, which primarily consists of four steps. These steps include random character generation, creation of Zhuyin annotated and Zhuyin-free images, injection of various degradation effects, and pairing them to generate the dataset. Each of the four distinct tasks contributes to creating a diverse and representative collection of document images suitable for training document enhancement algorithms.

The four components of the work are as follows:

### Character generation

The first step involves generating familiar Chinese characters, which are then randomly sampled to compose document sample images. The source of characters, the “Common Standard Chinese Characters Table,” is established by the Ministry of Education of the Republic of China (Taiwan). This table lists commonly used standard Chinese characters to standardize and regulate the glyphs of Chinese writing to enhance readability and aesthetics. These standard characters are curated by experts based on traditional calligraphy, linguistics, and philology to ensure they meet the demands of both traditional culture and modern writing.

The character table typically includes commonly used words approved by the Ministry of Education and undergoes regular updates and adjustments to reflect societal changes. It is applicable in educational settings for teaching and examinations and is widely employed across various sectors, such as publishing, printing, and advertising. Utilizing this standard character table ensures text consistency and standardization, enhancing communication effectiveness and cultural heritage preservation.

To reflect real-world scenarios of commonly used Chinese characters, all characters are selected from the 4,808 widely used characters listed in the “Common Standard Chinese Characters Table” published by the Ministry of Education of the Republic of China (Taiwan), as shown in Table 1. A random selection mechanism ensures equal probability for each character to be chosen. Each document sample comprises 96 randomly selected Chinese characters from the dictionary. In our dataset, each document image contains 96 randomly selected characters from the predefined Common Standard Chinese Characters Table. The selection process is implemented using a uniform random sampling method, ensuring that each character from the set has an equal probability of being chosen for every document. This random sampling is performed with replacement, meaning that the same character can appear multiple times both within a single image and across different images in the dataset. By using this method, we maintain the diversity of character combinations while ensuring statistical randomness in the generation of each document. This approach helps create a robust dataset that accurately reflects the variability of real-world documents, thus supporting effective training for document enhancement tasks.

**Table 1:** Example of word table with Unicode values and corresponding Traditional Chinese characters.

#	Unicode	Character
0001	U + 4E00	一
0002	U + 4E01	丁
0003	U + 4E03	七
0004	U + 4E09	三
⋮	⋮	⋮
4805	U + 9F72	齲
4806	U + 9F8D	龍
4807	U + 9F94	龔
4808	U + 9F9C	龜

### Zhuyin symbols

Zhuyin symbols, widely used in Taiwan and some overseas Chinese communities, serve various purposes, such as learning, teaching, text input, and word annotation. This unique system not only assists individuals in accurately pronouncing Chinese characters but also provides a standardized way to express their pronunciation, which is significant in language instruction and text processing.

Hence, to accommodate the diversity of contexts in which Traditional Chinese is used, including documents annotated with Zhuyin symbols, all Chinese characters in the dataset are accompanied by authentic Zhuyin symbols, adhering to the standards for Zhuyin annotation. For instance, Zhuyin annotations are positioned to the right of each Chinese character, and tone marks are placed to the right of the Zhuyin symbols. The process of integrating Zhuyin annotations into the text is streamlined using the H.T.Wang Free Fonts^26^, which automatically displays both the Chinese characters and their corresponding Zhuyin symbols. When this font is applied, the system associates each Traditional Chinese character with its appropriate Zhuyin symbol based on a predefined mapping. This mapping ensures that the base Zhuyin symbols for consonants and vowels, along with any necessary tone marks, are correctly positioned. The ‘H.T.Wang Free Fonts’ facilitates the display of the Zhuyin annotations directly to the right of the corresponding Chinese characters, adhering to the conventional format. By simply using this font in the document, the characters and their phonetic guides are automatically rendered together, eliminating the need for manual annotation. This method ensures uniformity and consistency across the dataset, while also adhering to the traditional standards for presenting annotated Chinese text.

### Variety of degradation

**Fig. 2:**
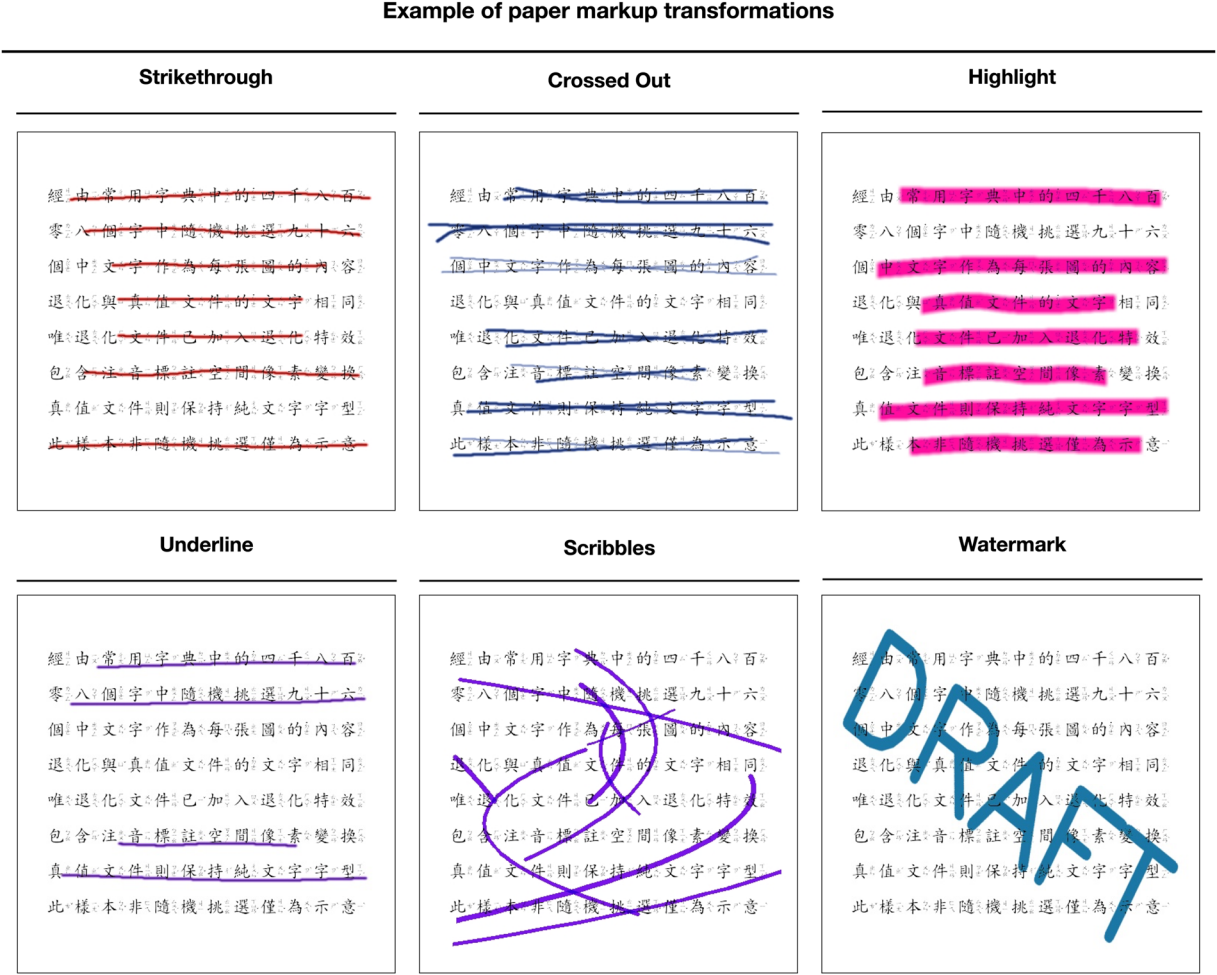
An example of paper markup applied to the document image. This transformation simulates the artificial annotations typically found on physical documents. Markup examples include strike through, crossed out, highlight, underline, scribbles and watermark that interfere with the legibility of the text, making the enhancement task more challenging.

**Fig. 3:**
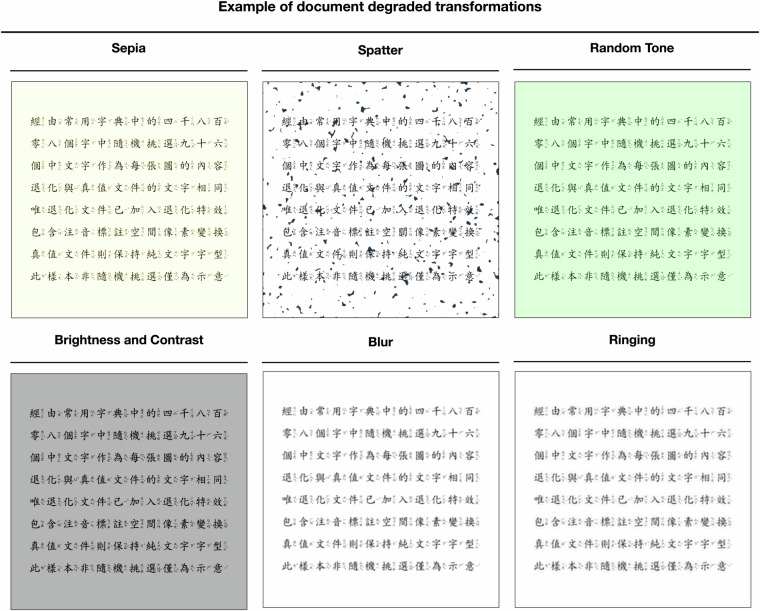
An example of a degraded document. This transformation simulates various types of document degradation commonly seen in real-world scenarios, such as sepia, spatter, random tone, brightness and contrast, blur, and tinging. These effects mimic the deterioration of paper documents due to aging, scanning artifacts, or environmental exposure, providing diverse and realistic data for training enhancement models.

**Fig. 4:**
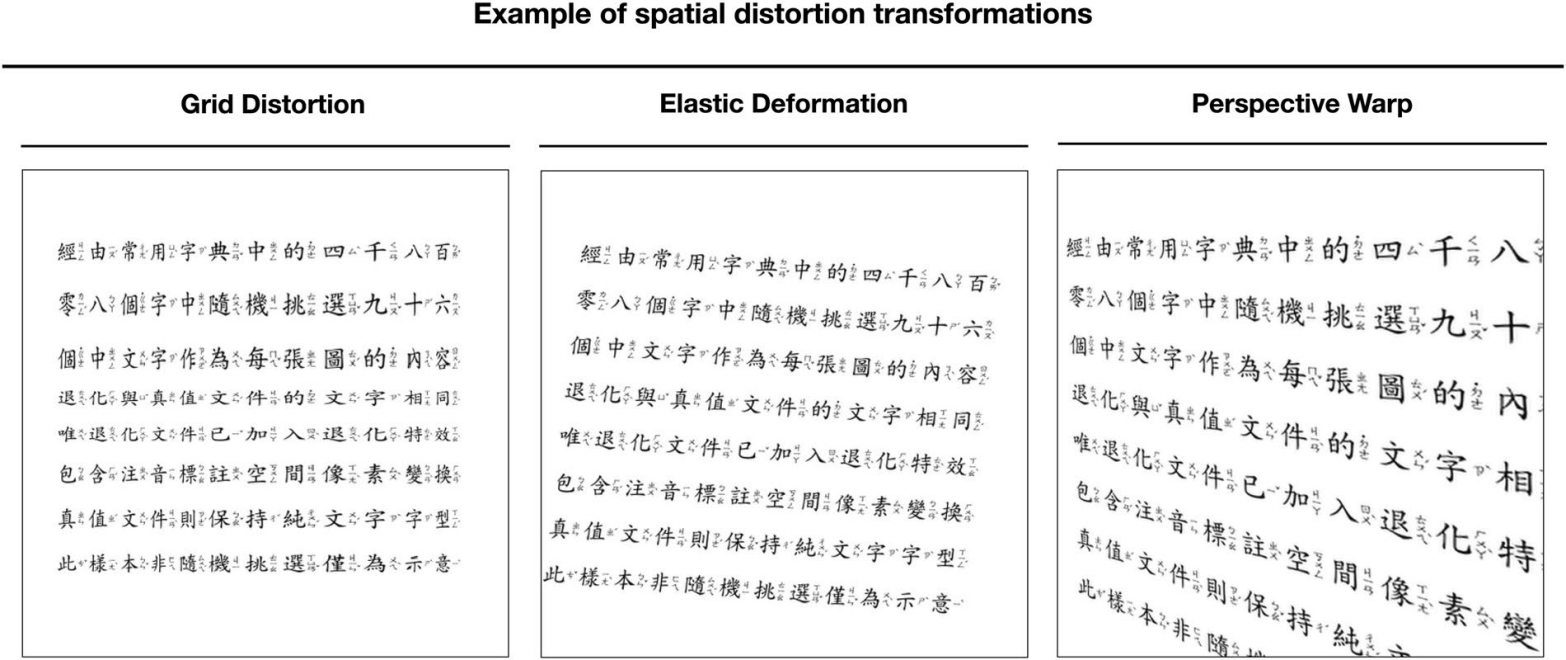
An example of spatial distortion applied to the document image. This transformation warps the spatial structure of the document by introducing geometric changes such as grid distortion, elastic deformation, and perspective warp. Spatial distortion mimics issues like misalignment during scanning or photographing documents, further challenging the model’s ability to restore the document correctly.

In the third work item, we synthesized a series of realistic degraded images commonly encountered in real-world document scenarios. We introduced three diversified degradation patterns into the dataset, as shown in Table 2. These include (1) **paper makeup**, representing human-made annotations on documents, as illustrated in Fig. 2. This type of degradation pattern mainly involves the marking behavior applied to documents by humans. This may include handwritten notes, markings, underlining, watermarks, etc., which are commonly found in real documents. By adding human-made markings to document images, we simulate the marking scenarios in real documents; (2) **document degradation**, depicting degradation due to natural or real-world factors, as depicted in Fig. 3. This type of degradation pattern is mainly caused by natural or real-world factors leading to the degradation of the document itself. For example, factors such as paper aging, exposure, water damage, etc., may lead to a decrease in document quality, affecting the readability and recognizability of the document. By simulating these natural degradation scenarios, we can better train models to handle these common degradation issues in the real world; and (3) **spatial distortion**, reflecting geometric changes in documents caused by factors such as shooting, transmission, and preservation, as shown in Fig. 4. This type of degradation pattern is mainly caused by geometric changes in the document during processes such as capturing, transmitting, and storing. This may include stretching, deformation, distortion, etc., which affect the geometric structure and shape of the document. By simulating these spatial distortions, we can better train models to handle the geometric changes commonly encountered in document capture and processing. To maintain sample diversity, these three groups are interconnected, with each group having a probability of occurrence set at $p\,=\,0.5$. Independently apply one of the following transformations—paper markup, document degradation, or spatial distortion—with a probability of $p\,=\,0.5$ for each. If a transformation is activated, one of its associated sub-transformations will be randomly selected and applied. For clear documents, no modifications are applied except for spatial distortion.

**Table 2:** An overview of the three main types of degradation patterns introduced into the JVZY dataset.

Variety of degradation	
Paper makeup	Parameter (with Augraphy^28^)
Strikethrough	strikethrough(num_lines_range = (2, 7),markup_length_range = (0.5, 1), markup_thickness_range = (1, 2))
Crossed Out	crossed(num_lines_range = (2, 7),markup_length_range = (0.5, 1), markup_thickness_range = (1, 2))
Highlight	highlight(num_lines_range = (2, 7),markup_length_range = (0.5, 1), markup_thickness_range = (1, 2))
Underline	underline(num_lines_range = (2, 7),markup_length_range = (0.5, 1), markup_thickness_range = (1, 2))
Scribbles	scribbles(scribbles_size_range = (100, 500), scribbles_count_range = (1, 6), scribbles_lines_stroke_count_range = (1, 2),scribbles_thickness_range = (2, 6))
Watermark	watermark(watermark_word = “random”,watermark_font_size = (6, 8), watermark_font_thickness = (20, 25), watermark_rotation = (0, 360))
**Document degraded**	**Parameter (with Albumentations**^29^**)**
Sepia	ToSepia(always_apply = False)
Spatter	Spatter(mean = (0.65, 0.65), std = (0.3, 0.3), gauss_sigma = (2.0, 2.0), intensity = (0.6, 0.6), cutout_threshold = (0.68, 0.68))
Random Tone	HueSaturationValue(hue_shift_limit = 20, sat_shift_limit = 30, val_shift_limit = 20)
Brightness and Contrast	RandomBrightnessContrast(brightness_limit = (−0.2, 0.2), contrast_limit = (−0.2, 0.2), brightness_by_max = True))
Blur	AdvancedBlur(blur_limit = (3, 3), sigmaX_limit = (0.2, 1.0), sigmaY_limit = (0.2, 1.0), rotate_limit = (−90, 90), beta_limit = (0.5, 8.0), noise_limit = (0.9, 1.1))
Ringing	RingingOvershoot(blur_limit = (7, 15), cutoff = (0.7, 1.57))
**Spatial distortion**	**Parameter (with Albumentations**^29^**)**
Grid Distortion	GridDistortion(num_steps = 5, distort_limit = (−0.3, 0.3), interpolation = 4)
Elastic Deformation	ElasticTransform(alpha = 1.0, sigma = 50.0, alpha_affine = 50.0, interpolation = 4)
Perspective Warp	Perspective(scale = (0.05, 0.1), keep_size = 1, interpolation = 4)

These patterns are designed to simulate common degradation scenarios encountered in real-world document images.

### Paired dataset

The dataset is curated by synthesizing original high-quality document images alongside their corresponding degraded versions, with meticulous control over the degradation process is applied to the document images to ensure both realism and variety within the dataset. Each original document image is paired with its corresponding degraded version to form a paired dataset. Such pairings are instrumental in training image-to-image generative models to learn the mapping between original document images and their degraded counterparts, enabling document image restoration and enhancement.

The final format of the paired dataset consists of two groups of JPEG images: ground truth images and their corresponding degraded versions. Each image is saved with a resolution of 512 × 512 pixels. The resolution of 512 × 512 pixels was selected for the paired dataset based on a balance between computational efficiency and visual clarity. Through previous empirical experimentation, we determined that this resolution is sufficient to capture the critical features of Traditional Chinese characters, which are often complex in structure. Despite the inherent intricacies of these characters, this image size allowed the U-net-based document enhancement model to successfully learn the mapping between degraded and clean images. This resolution was also chosen with the practical considerations of model training efficiency and dataset scalability in mind. The size ensures the dataset is computationally manageable, especially for large-scale deep learning tasks, while maintaining high relevance for real-world document enhancement scenarios. However, we recognize that specific applications may require higher granularity, particularly for tasks involving very fine details in complex characters, and may benefit from higher-resolution images. As such, future iterations of the dataset could provide higher-resolution options or the flexibility to adjust the resolution to suit specific use cases.

For instance, an image pair might be labeled as JVZYpair_0_00001_zhuyin-free.jpg for the clean image and JVZYpair_0_00001_zhuyin.jpg for its degraded counterpart. This standardized naming convention ensures that each pair can be easily identified and used in model training.

## Data Records

The dataset consists of 10,000 ground truth JPEG images and 10,000 degraded JPEG images. Each image has a resolution of 512 × 512 pixels and contains 96 randomly selected Traditional Chinese characters, totaling approximately 1,920,000 characters in the entire dataset.

### JVZY dataset

All synthetic document images are available on Zenodo (10.5281/zenodo.10963646^27^). The image files follow a specific naming convention:

- Ground truth images: <DNV> _ <BGV> _ <PNN> _ <PNV>.jpg, for example: JVZYpair_0_09999_zhuyin-free.jpg, and
- Degraded images: <DNV> _ <BGV> _ <PNN> _ <PNV> .jpg, for example: JVZYpair_0_09999_zhuyin.jpg,

where <DNV> denotes the name and version of the dataset, <BGV> indicates the background image version, <PNN> represents the pair number, and <PNV> signifies the pair name.

## Technical Validation

**Fig. 5:**
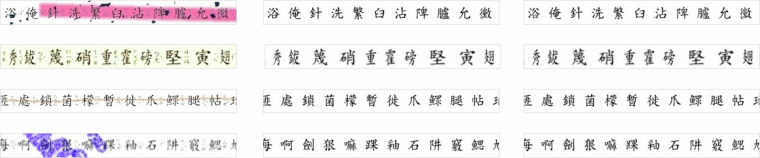
Qualitative document enhancement results using the U-net model. The figures show the input degraded images, corresponding ground truth, and the results after processing with the U-net model. The visual inspection ensured that the degradation is consistent with clean documents and remains within the bounds of common sense. These results demonstrate the model’s ability to effectively restore degraded document images, validating the dataset’s quality and suitability for document enhancement tasks.

To validate the dataset’s quality, we conducted two types of evaluations. First, the team performed visual inspections on each image to ensure that no anomalous characters were generated, that the degradation matched the text of clean documents, and that the level of degradation did not exceed the bounds of common sense. Second, we trained a document enhancement model using the dataset to verify its effectiveness. The model utilized an image-to-image architecture based on the U-net model. Here are the qualitative document enhancement results generated by the U-net model. Figure 5 depicts the qualitative document enhancement results generated by the U-net model. They include the input degraded image, the ground truth, and the result after processing with the U-net model. The Table 3 shows the average metric values calculated across 1000 paired images from the validation set, which is used to assess the performance of the U-net model for document enhancement. Each metric offers a different perspective on how well the model is performing in terms of restoring degraded document images. These include MAE (Mean Absolute Error), MSE (Mean Squared Error), RMSE (Root Mean Squared Error), PSNR (Peak Signal-to-Noise Ratio), and SSIM (Structural Similarity). Each of these metrics provides valuable insights: while MAE and MSE evaluate pixel-level errors, PSNR and SSIM focus on visual quality and perceptual similarity, and RMSE provides an overall error measure in the same units as the original data.

**Table 3:** Average metric values for the 1000 paired validation images.

Metrics	Value
MAE	2.698
MSE	124.139
RMSE	7.593
PSNR	28.747
SSIM	0.979

## Usage Notes

All image files are bundled into two compressed ZIP files named JVZY_images_ <PNV> .zip for quicker and more convenient downloads. Upon downloading the files, users interested in employing them for deep neural networks or other machine learning models capable of image-to-image training can utilize the accompanying script^27^. This script facilitates the generation of training and validation sets, as well as training a baseline U-net model, as needed.

## Data Availability

All code is available in Zenodo (10.5281/zenodo.10963646^27^).
